# Femicide in the United States: a call for legal codification and national surveillance

**DOI:** 10.3389/fpubh.2024.1338548

**Published:** 2024-02-28

**Authors:** Patricia C. Lewis, Nadine J. Kaslow, Yuk Fai Cheong, Dabney P. Evans, Kathryn M. Yount

**Affiliations:** ^1^Department of Health Sciences, Sacred Heart University, Fairfield, CT, United States; ^2^Department of Psychiatry and Behavioral Sciences, Emory University, Atlanta, GA, United States; ^3^Department of Psychology, Emory University, Atlanta, GA, United States; ^4^Hubert Department of Global Health, Emory University, Atlanta, GA, United States; ^5^Hubert Department of Global Health, Department of Sociology, Emory University, Atlanta, GA, United States

**Keywords:** femicide, violence against women (VAW), intimate partner violence (IPV), homicide, United States, gender

## Introduction

Femicide refers to the intentional gender-related killing of women and girls (1). Despite the high prevalence of female murder victimization in the United States (U.S.) (2, 3), the U.S. lags behind other nations in defining and documenting gender-related female homicides (4). While efforts are underway within the criminal justice and public health sectors to better track violent deaths, deficient surveillance systems limit efforts to estimate the annual incidence of femicide in the U.S. Here, we position femicide as a preventable death that should be treated as a social and public health problem and a distinct form of homicide in the legal code. This approach is especially salient, given the documented increase of non-lethal intimate partner violence (IPV) in major cities (5) and nationally (6) during the COVID-19 pandemic, demonstrating the collateral impacts of public-health crises on violence against women (VAW).

## Making the invisible visible: efforts to name, define, criminalize, and document femicides in the U.S.

Feminist sociologist Diana Russell coined the term femicide in her testimony about misogynist murder before the 1976 International Tribunal on Crimes against Women (7). The act of naming by Russell and other scholars and activists brought femicide to the forefront of international movements to stop VAW (8). Yet, most countries, including the U.S., lack a legal definition of femicide, complicating its surveillance, and by extension, prevention and response (9). Countries throughout Latin America have led the way to criminalize femicide through legal statutes that mandate accountability (10). The U.S. does not have a separate penal code for gender-related killings (4), making it difficult to track femicides. According to the U.S. Federal Bureau of Investigation (FBI), an estimated 4,970 female victims were murdered in 2021, one third of whom were documented to have been killed by an intimate partner (2). This is likely an underestimate, as municipal reporting to the central system is not mandatory (6, 11, 12) and data from <63% of police agencies were included in the 2021 report (2). Other estimates utilizing multiple data sources suggest that half of female victims of homicide in the U.S. are killed by intimate partners (13, 14). Importantly, reports of women being murdered are not always categorized as a homicide (15), and the motivations for a homicide and the victim's relationship to the perpetrator often go undocumented (16).

## Calls for action on femicide in the U.S.

According to the World Bank (3), the U.S. ranks 34th worldwide for the intentional murder of females. Yet, such crimes are not categorized as femicides in the penal code, making it difficult to classify and to track the gender-based murder of women and girls. Lacking a clear legal definition of femicide in the U.S. and a surveillance system that identifies and classifies these murders accurately, such acts may appear isolated, hiding the scope of the problem and limiting public health prevention and legal response. Drawing from our experience researching VAW in the U.S. (5, 17, 18) and lessons learned from countries in Latin America (10, 19) and the UK (20–22), we call upon U.S. policy makers to implement three *urgent actions* regarding the legal conceptualization and surveillance of femicide data in the U.S.: (1) including a clear, comprehensive definition of femicide in the penal code; (2) improve the accuracy and completeness of data on femicide including perpetrators; and (3) increase the ability to disaggregate data on femicides to account for intersectional identities, for example, on the bases of race or ethnicity, class, country-of-origin, gender identity, and sexual orientation.

### Include a clear, comprehensive definition of femicide in U.S. penal code

Building upon the efforts of feminist movements and other country contexts (10, 23) the U.S. can move toward improved surveillance capacity by adopting a definition that harmonizes with existing ones, allowing us to move toward global surveillance capacity. Femicide often is defined as gender-related killing of women and girls (1, 23) and is considered an extreme violation of a woman's right to self-determination (9), depriving her of fundamental human rights to life and bodily integrity, as protected by international law (24). While femicide takes multiple forms, the phenomenon often is grouped into: (1) *intimate femicide*—femicides perpetrated by a current or former intimate partner and (2) *non-intimate femicide*–familial femicide, human-trafficking-related femicide, sexual femicide, and crime-related femicide among others (1, 25). Including and clearly defining multiple forms of femicide in a legal definition is “essential to give visibility to the many forms of gendered killings” (10).

We call on the U.S. to follow the example of the Inter-American Model Law on the Prevention, Punishment and Eradication of the Gender-Related Killing of Women and Girls (Femicide/Feminicide) (19) and reform the penal code to include femicide as a form of aggravated homicide as has been done in Argentina, Brazil, and Uruguay (10). Modifications to U.S. criminal statues including a separate category for femicide and clear definitions of its multiple forms would facilitate classification of the gender-related killing of women and girls as femicides, setting critical groundwork for improved surveillance.

### Improve accuracy and completeness of data on femicide including perpetrators

To catalog and analyze femicides under the new proposed penal code, existing data structures also need to be updated. Of note, the dynamics of female homicides differ from male homicides (13, 26), including that most female homicides take place in the private sphere (26, 27). As such, contextual information about each homicide is critical. Following guidance from the UN Office on Drugs and Crime on a statistical framework for measuring femicide (28) and research on male-perpetrated female homicides in Canada (23), we recommend that sex/gender-related motives/indicators (SGRMIs) be cataloged and assessed to ascertain whether the killing was a femicide. SGRMIs are characteristics that indicate whether the homicide was “rooted in perpetrators' misogynistic attitudes” (23) and can include factors such as current or past intimate relationship with the perpetrator, familial relationship, perpetrator history of IPV, evidence of sexual violence accompanying the killing, victim experienced human trafficking or involvement in sex work, bodily mutilation and/or public exposure, and evidence of the killing being motivated by hatred of women (23, 26, 28). Accurate and timely collection of the following additional contextual data is necessary to determine the gender-related nature of the killing: gender identity and sexual orientation of the victim, pregnancy status of the victim, perpetrator's history of restraining orders, economic activity status of the victim and perpetrator, and gender-related motive for the killing (28). Currently, the U.S. does not have a surveillance system that collects comprehensive information in these categories for all murder cases. The establishment of a review board, much like the domestic homicide reviews in the UK (20, 22), would be required to collect such data and determine whether a femicide has occurred.

Data on deaths in the U.S. exist under the aegis of public health and criminal justice surveillance systems. In the former, death due to assault can be tracked using vital statistics data from the U.S. Centers for Disease Control and Prevention (CDC) WONDER database. While violent female deaths/homicides are identifiable in these data, they provide limited information about the perpetrator (29). Criminal justice surveillance historically took place through the Uniform Crime Report (UCR), where the Supplementary Homicide Reports included contextual data. In 2021, the UCR was replaced with the National Incident-Based Reporting System (NIBRS), however only 66% of police agencies reported crimes to the new system in 2022 (17, 30), which is similar to the problem with the prior system (12).

The National Violent Death Reporting System (NVDRS), hosted by the CDC's National Center for Injury Prevention and Control, began tracking violent deaths in the U.S. in 2003. This surveillance system compiles facts from death certificates, coroner/medical examiner reports, law enforcement reports, and toxicology reports into one database (31). Where available, the NVDRS includes contextual information about the murder including the relationship between the victim and perpetrator (29, 32). Currently, the NVDRS collects data from 48 states, District of Columbia, and Puerto Rico. While the NVDRS provides the necessary linkages between criminal justice and public health data, the reporting of SGRMIs is not mandatory and known community-level correlates of non-lethal forms of VAW—such as neighborhood poverty, residential instability, and gender inequality (33–35)—are not tracked.

**Figure 1:**
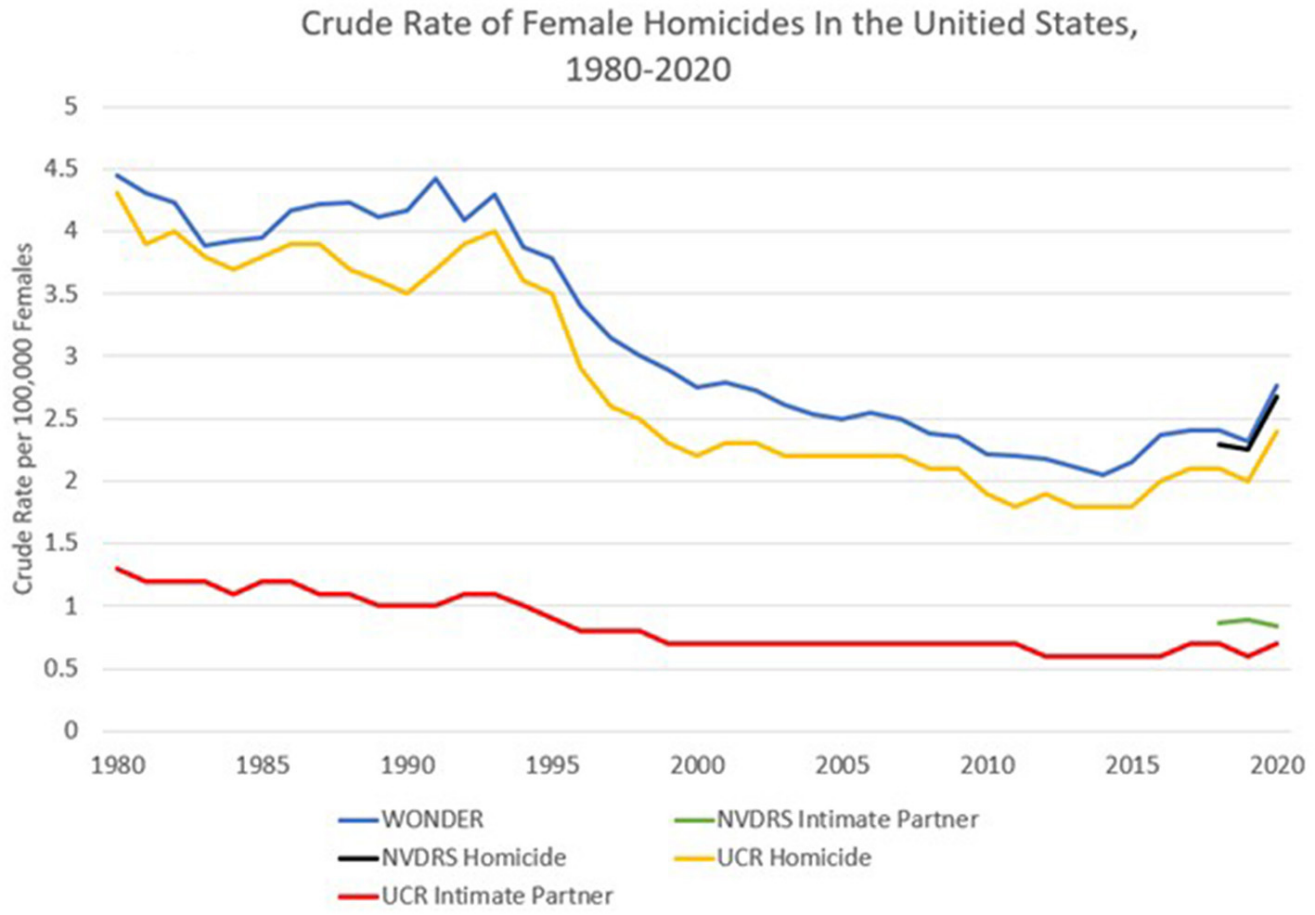
WONDER: the CDC's WONDER mortality database. Data includes all homicide victims categorized as females and were captured from the following files: “Compressed mortality, 1968–1978” using ICD-8 codes, E960-E969; “Compressed mortality, 1979–1998” using ICD-9 codes E960-E969; “Compressed mortality, 1999–2016 using ICD-10 codes Y87.1, X85-Y09; “About underlying cause of death, 2018–2021” using ICD-10 codes X85-Y09. NVDRS intimate partner: the national violent death reporting system (NVDRS). Data includes all homicide victims categorized female that had a reported intimate relationship with the perpetrator (current or former spouse, boyfriend/girlfriend or common-law partner). Importantly, the NVDRS data from 2018 excludes data from Arkansas, Hawaii, Idaho, Mississippi, Montana, North Dakota, South Dakota, Tennessee, Texas, and Wyoming. NVDRS data from 2019 excludes data from Arkansas, Idaho, Mississippi, New York, South Dakota, Tennessee, and Texas. NVDRS Homicide: NVDRS data on all female homicides, regardless of relationship to perpetrator. The same state exclusions apply as above. UCR homicide: the federal bureau of investigation's (FBI) uniform crime reporting (UCR) program's supplementary homicide reports (SHR). This data includes all reported homicide victims categorized as female from 1980 to 2020 regardless of relationship to perpetrator. UCR intimate partner: UCR data on all reported female homicides that were indicated as perpetrated by current or former intimate partner (spouse, boyfriend/girlfriend or common-law partner).

Figure 1 compares the tracked murders of females over time (1980–2020) using data from the CDC WONDER database and the FBI'S UCR. Also included are NVDRS data beginning in 2018, when at least 40 states were included in data collection. The trend lines indicate that the public health system (WONDER) consistently captures more female homicides than does the crime tracking system (UCR). While the crude rate of reported female homicides has decreased since the 1980s, an uptick in female homicides appeared in 2020 during the COVID-19 pandemic, which corroborates other studies (5, 17). Regarding intimate partner homicides, UCR data indicate that intimate partners perpetrate about one third of all female homicides; however, a substantial percentage of female homicides are missing information on victim-perpetrator relationship. Notably, for the years available, NVDRS data indicate higher rates of intimate partner homicides than captured in the UCR data. Finally, the gender identities of the victims are not reported, so some subgroups (e.g., transgender women) may be missing or subsumed into an ascribed category, rendering them invisible.

The NVDRS (black line in Figure 1) is the most comprehensive system to track femicides, and we propose that SGRMIs be included as mandatory fields in homicide reports. We also recommend linking the NVDRS homicide data with census data to detect community-level risk factors. Following the Inter-American Model Law on the Prevention, Punishment and Eradication of the Gender-Related Killing of Women and Girls (Femicide/Feminicide) (19), we further propose that improved NVDRS data be used to establish a femicide observatory that catalogs all cases of killings of women. Several other countries have successfully established femicide observatories; such observatories, run by civil society organizations play an important watchdog role providing contextual and nuanced analysis which supplements national data sources (36).

### Improve ability to disaggregate femicides by intersectional identities

While an urgent need for improved accuracy in femicide data exists (17, 23, 37), once an adequate surveillance system is in place, questions on risk patterns must be addressed. Crenshaw (38) has emphasized the need to situate women's experiences of violence at the intersection of multiple social hierarchies. Thus, the ability to disaggregate data on femicide by marginalized social identities is critical to identify intersectional risks and impacts and to direct resources to the most vulnerable.

For example, recent research using the NVDRS, despite its limitations, indicates that indigenous women have higher rates of homicide victimization than all other ethnic groups (39). This finding prompted an executive order to address the crisis of missing and murdered Indigenous peoples (40). Black women in America also face a greater risk of being murdered (39, 41), particularly during pregnancy (42). The higher rates of homicide for Indigenous and Black women indicate that the murder of women of color takes place at the intersections of racism and sexism (43). Concerning country of origin, research suggests higher rates of homicide victimization among U.S.-born individuals; however, certain foreign-born groups, including those from Honduras, El Salvador, and Jamaica, have higher than average homicide victimization (44). Notably, these countries have high rates of homicide amidst complex historical legacies of colonialism, slavery, civil conflict, and weak governance (45). Data from Europe indicate that citizenship status is a risk factor for female homicide (46). To our knowledge, female homicide victimization by class or income has not been examined with NVDRS data. Finally, as gender identity and sexual orientation are not included in the aforementioned public surveillance data, the intersectional vulnerabilities of LGBTQ+ people to identity-related homicide are unknown at a national scale. The ability to disaggregate female homicides by other marginalized identities may inform more refined definitions of identity-motivated deaths in the penal code.

## Conclusion: recommendations call to action

In the U.S., a clear definition of femicide is lacking, as is a surveillance system capable of identifying and classifying gender-related murders with attention to intersectional vulnerabilities. To address these gaps, we call on policymakers to (1) include a clear definition of femicide and its various forms in the U.S. penal code; (2) improve the accuracy and completeness of data on femicide including information on perpetrators; and (3) make documentation of expanded SGRMIs mandatory to allow for disaggregation of data on femicides according to other intersectional vulnerabilities. Practical steps would include a consensus process to inform a legal definition of femicide that draws on international successes, piloting changes to femicide surveillance systems in an initial handful of states, and with better data, estimating the costs of femicide to individuals, families, vulnerable communities, and society to clarify the benefits of prevention. Methodologically sound data ensures greater accuracy, validity, and reliability, which not only underpins rigorous research on femicide, but also aids in the creation of effective legal policies.

Each proposed change is critical for quantifying the incidence and costs of femicide and developing focused and life-saving prevention strategies so that femicide is no longer a major public health problem in the U.S. or globally. In addition, pertinent legislation is essential, such as ongoing authorization and associated appropriations for the violence against women act and U.S. National Action Plan to End Gender Based Violence (47), so there are improved prevention and intervention programs, more consistent application of evidence-based practices by law enforcement, and the requisite support for law enforcement to investigate and prosecute cases.

## Author contributions

PL: Conceptualization, Methodology, Project administration, Visualization, Writing—original draft, Writing—review & editing. NK: Conceptualization, Funding acquisition, Writing—review & editing. YC: Methodology, Writing—review & editing. DE: Supervision, Writing—original draft, Writing—review & editing. KY: Conceptualization, Funding acquisition, Project administration, Supervision, Writing—original draft, Writing—review & editing, Methodology.
